# Visualisation and Interpretation of Student Strategies in Solving Natural Science-Based Tasks Using the Eye-Tracker

**DOI:** 10.16910/jemr.11.4.4

**Published:** 2018-08-31

**Authors:** Jana Škrabánková, Lukáš Laš, Petr Bujok

**Affiliations:** University of Ostrava, Ostrava, Czechia; www.osu.eu

**Keywords:** Eye-tracking, data analysis, experimental study, students gifted in natural science, assignment solving strategies, areas of interests

## Abstract

This paper presents a research on students using the Gazepoint device to visualise the prac-tices and strategies that students used in order to solve assignments in the disciplines of natural science. The analysis of visual perception of students is complemented by a ques-tionnaire survey for a group of respondents aged 15-16. The essence of the study was to find out how the students proceeded in monitoring assignments displayed on the screen, how they continued working with the assignments, and whether the layout of the schematics, tables and applied images affected students ‘correctness for the solution. The main aim of the research was to find some similar segments in the experimental data and obtained clus-ters that would suggest a similar approach of problem solving by students – respondents, and to find out if, and possibly how, some strategies in the assignments differ for the talented students from the standard pupil population and compare the outcomes with students’ char-acteristics. The other aim of study was to confirm the presence of gifted students in natural sciences in a given sample of respondents on the basis of eye-tracking technology. Also on the basis of similarities in assigned task solving the aim was to find other students who can be seen similarly to the gifted ones from a view of e.g. physiological dynamics of eyes of the students in the context of the given selected seven tasks in the area of the chemical elements identification. For both groups of students, some basic measures are proposed to increase the efficiency of students‘ work with an assignment displayed on a computer screen. Our results show that in the task solving, one gifted student was identified next to a cluster of four similarly performing students on the basis of eye-movements parameters.

## Introduction

The designation a “gifted” (or talented) person is commonly used in interpersonal communication in different contexts often without deeper reflection on the consistency or the differences of both concepts. It must be admitted that there is no unambiguous and generally accepted interpretation of when an individual is labelled “gifted” or “talented” (here the two adjectives are understood as synonyms). This is valid both for Europe and the world. Some experts identify these terms in general and consider them synonymous; others distinguish between them and perceive them as a certain sequence. Gagne (1) is up today one of the few experts in the field of giftedness who tries to terminologically distinguish between the notions of giftedness and talent. Under the giftedness, he designates the innate abilities related to psychic functions. The giftedness is transformed into the talent in the course of development, and this talent relates to a specific area of human activity. As can be seen, there is a small point in arguing over the twofold understanding of the concept, since such a polemic has been going on for some time. Moreover, to a certain extent, it diverts the attention from the essence of the problem, i.e. it keeps dealing with the same individual. Most of the attention is usually given to a type of the so-called cognitive (also intellectual) talent. We tend to use the term intellectual giftedness, as the term intellect rather than the concept of cognition serves to impair the functional integration of individual intellectual abilities, including cognitive abilities and skills.

The issues of talent and gifted students have also been gradually gaining momentum in Czechia. The Ministry of Education, Youth and Sports of the Czech Republic considers the support and development of gifted students, particularly in the fields of natural science and technology, one of its main priorities. The important official step for the support to the talented students was the Decree No. 27/2016 Coll., whose topicality testifies a social need for the support of gifted students in the Czech educational system. There is a fundamental change from this decree in contrast to the previous decree, and that is the definition of the so-called extraordinarily-gifted student. While the definition of a gifted student remains with respect to the previous Decree No. 72/2005 Coll. unchanged, newly the term “extraordinary gifted student” has appeared. The new Decree of 2016 states that:

“***Gifted and extraordinary gifted pupil***

(1) For the purposes of this Decree, a gifted pupil is considered to be a pupil, who under adequate support comparing to peers, shows a high level in one and more areas of intellectual ability, in movement, manual, artistic or social skills.

*(2) For the purposes of this Decree, an extraordinary gifted student is considered to be primarily a pupil, whose distribution of abilities reaches exceptional levels at high creativity in a whole range of activities or in individual areas of intellectual abilities, in movement, manual, artistic or social skills.”* (2)

What makes such a significant difference? What can teachers rely on in work with an extraordinary gifted student? It is the formulation of point 2) *“... reaches exceptional levels at high creativity in a whole range of activities or in individual areas.*” This statement eliminates some myths about gifted students, for instance, that a gifted student can always do everything, that a gifted student actually is a well-taught pupil, so s/he doesn’t need teachers’ support and s/he gets along alone. However, the opposite is true. In this article, we have attempted to uncover possible differences in the solution of tasks by gifted students.

It is generally understood that gifted students constitute two to three percent of the population. Based on respectable foreign studies, Joan Freeman: the British psychologist writes that if all children had the necessary conditions for their development, 20-25% of them would be able to excel in some area. (3) This means that those students with whom one counts as with the gifted make only an imaginary tip of the glacier and the vast majority of potentially gifted individuals remain unidentified and not properly developed. If luckier students are assigned to a group of the gifted students in natural sciences (here belong students gifted in mathematics, physics, chemistry, and biology), it is possible to work further with them appropriately. However, gifted students in natural sciences do not necessarily have to be gifted in all of subjects at the same time. It often concerns a certain combination of talents in some subjects, which is complemented by the general interest of students in science as a whole.

According to American psychologist Howard Gardner (4), the talent for natural science is related to the natural science intelligence. Gardner depicted this intelligence in the theory of multiple intelligences and expanded it with an eighth item in 1996, the natural intelligence. She characterized this intelligence as *“the ability to observe, understand and classify the natural entities. A natural scientist becomes the one who can, easily and better than others, recognize and classify plants, animals and non-living objects (including life at the molecular level) and perceive their links with the environment.”* (5).

Therefore, we attempted to use the Gazepoint eye-tracker in order to verify talents in natural sciences at four students who were part of a group of 26 respondents, working on seven tasks in the area of the physical chemistry (see Appendix 1). The group of four gifted students was nominated in advance on a basis of the comparison of experimental results from a pedagogical-psychological advisory office, and on the nomination of students by a teacher with experience with education of the gifted students. It was also considered that we would find latent talent in natural sciences at someone from the other respondents. We were curious about how the two groups of students progressed in work with text and images presented on a monitor screen and how these groups were then able to solve assigned tasks (6, 7, 8).

Legend of the experiment: The respondents were not limited by time in task solving. The transition between two consecutive tasks was conducted by a technician staffer, based on a request of a given respondent. After transition to the next task, it was not possible to return to the previous task. The laboratory conditions were kept in course of the experiment (9).

Differences in eye movements of school-age students as they solve tasks of different levels of difficulty in the natural sciences (computer science, mathematics, physics, and biology) were studied previously elsewhere (10). Given authors assumed that the fixation duration average can be taken as an index of the difficulty level of the task being solved.

Regressions between recordings of pupil size and fixation disparity were studied to allow correcting the pupillary artefact (11). The findings provide a quantitative estimation of the pupillary artefact on measured eye position as function of viewing distance and luminance.

Studies on applications of eye-trackers have been increasing in scope, e.g. studies on eye movements in reading and other information processing tasks, such as music reading, typing, visual search, and scene perception, to mention few (12). Studies were done also on eye-tracking in student problem solving (13). We focus on problem solving in natural science education, more specifically in physical chemistry assignments, using the eye-tracking approach.

The eye-tracking technology has gradually taken attention in natural science education (14, 15, 16). It is also the case of eye-tracking in chemistry education research (17, 32, 18), etc., the case of eye-tracking in physics education research(19, 20, 21).

Visual model representation and its importance in chemistry education was one of the reasons for using eye-tracker to bring more insights to this topic (18). A multimodal data approach to chemistry education research using eye-tracking was presented by Hansen (22), based on study on college chemistry general students mapping their metavisualisation skills. The key role of graphical representation of data in learning physics was presented by Susac et al. (19), when students who had visual representations of physical data scored better and faster than those who didn’t.

Tracking gaze in chemistry education in order to identify learning strategies was attempted by Peterson et al. (32). Studying molecular representations within organic chemistry classes was picked up by Plekker & VandenPlass (23). Their results excluded gender-related bias and confirmed experience as a factor of correctness.

### Research question and methods

The main aim of the research was to find some similar segments in the experimental data and obtained clusters that would suggest a similar approach of problem solving by students – respondents, and to find out if, and possibly how, some strategies in the assignments differ for the talented students from the standard pupil population and compare the outcomes with students’ characteristics. The other aim of study was to confirm the presence of gifted students in natural sciences in a given sample of respondents on the basis of eye-tracking technology.

The Gazepoint eye-tracking system was used in our research. It is exactly designed for academic researchers and usability and UX designers. The system is affordable and works very well for this type of application. The Gazepoint GP3 HD UX Bundle is the most popular UX testing product currently. This bundle includes the GP3 HD & Gazepoint Analysis UX Edition software. (24) The parameters of the device were set in recommended values.

**Fig. 1:**
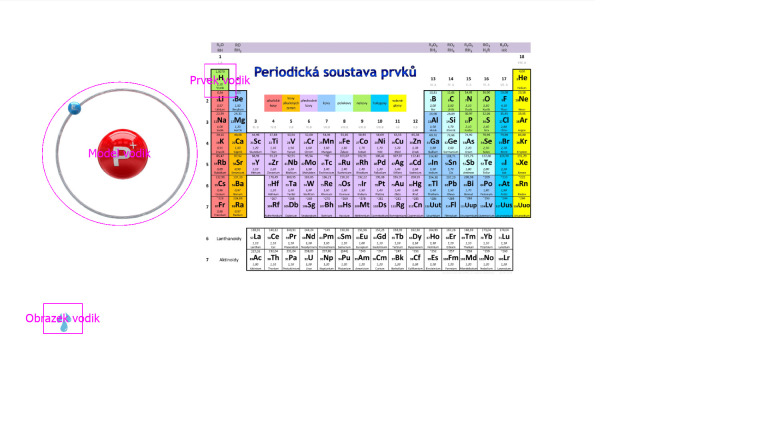
Designation of AOI for hydrogen

We displayed the measured data in the program OGAMA (25) and used the mode - Areas of Interest (AOI). In this mode it is possible to draw the AOI of different shape and size into a studied image. In each of the images we presented to the respondents on the screen, we have identified three AOI - a model scheme of the atom, an area of position of a given element in the Periodic System of Elements, and a hint image (see Figure 1). Then we used the options for displaying the data of an individual student (or a given group of students, or all persons) that we interpreted by pedagogical-psychological methods.

We selected these areas in accordance with the didactic analysis of the image, because the key to solving all the chemical problems was:

-
  whether the respondent is involved in the mechanical counting of electrons in the model scheme of the atom,
-
  whether the respondent focuses on the area location of a given element in the Periodic System of Elements,
-
  whether the respondent uses the hint image to solve the task.

Using the AOI comparison in a particular image, we also tracked:

-
  the logical solution procedure,
-
  whether the respondents verified the solution (i.e. whether the respondents gave back a look at the AOI).

The system allows to display either the total fixation time in a given AOI or the number of fixations, as well as the absolute/relative transitions between the individual AOI. It is possible to export the data in a text file using the “export AOI data” option. The analysis was performed in the NCSS program (29). For the sake of completeness of our findings, we have completed our research with a questionnaire survey.

## Results of research

There were 26 students with various talents. The talents were measured in 7 tasks from the field of the physical chemistry (Table 1).

**Table 1.** The list of the tasks, solving by students

U1	Vodik.jpg	Hydrogen
U2	Kremik.jpg	Silicon
U3	Kyslik.jpg	Oxygen
U4	Uhlik.jpg	Carbon
U5	Dusik.jpg	Nitrogen
U6	Zlato.jpg	Gold
U7	Lithium.jpg	Lithium

For each student (and assignment), the success (Binary number) and some eye features were measured (by eye-tracking metrics). For the purpose of our research, we identified selected variables (Table 2). In this report, some statistical methods are applied to show how various features influenced the talent of the students.

For better description of the results, original names of the variables are abbreviated:

**Table 2.** Selected variables for data analysis

st	StartingTime(ms)
du	Duration(ms)
fc	GazeFixationsCount
fs	GazeFixationsPerSec
sl	GazeAverageSaccadeLength(px)
sv	GazeAverageSaccadeVelocity(pxps)
cl	GazeFixationConnectionLength(px)
pv	GazePathVelocity(pxps)

Table 3 shows basic characteristics of students’ successes over all tasks based on gender. The number of successfully solved tasks for 11 male students is slightly higher (50) compared with 12 female students (49).

**Table 3.** Success of the students in dependency on gender

success	sum	count	mean	mean (%)
female	49	12	4.1	58.3
male	50	11	4.6	64.9

Starting Time (ms) indicates the time of the beginning of the assignment, it depends on the time of processing the previous tasks.

Duration (ms) is a time period for the assignment resolution, the longer it lasts, the longer the student has spent the time over the assignment.

Gaze Fixations Count - the number of eye scanpath lengths in the image. More overall scanpath lengths indicate less efficient search (perhaps due to sub-optimal layout of the interface). (26).

Gaze Fixations PerSec - the number of eye scanpath lengths on the frame per second. A longer fixation duration indicates difficulty in extracting information, or it means that the object is more engaging in some way. (27).

Gaze Average Saccade Length (px) – average duration of the saccade (transition between eye movement scanpath lengths on the frame in pixels).

Gaze Average Saccade Velocity (pxps) - average velocity of the saccade in pixels per second, i.e. the length of all saccades / time span of values.

Gaze Fixation Connection Length (px) - length of the “path” between eye scanpath length. A longer scanpath indicates less efficient searching (perhaps due to a sub-optimal layout). (28).

Gaze Path Velocity (pxps) – eye velocity over the “path” in the image.

**Fig. 2:**
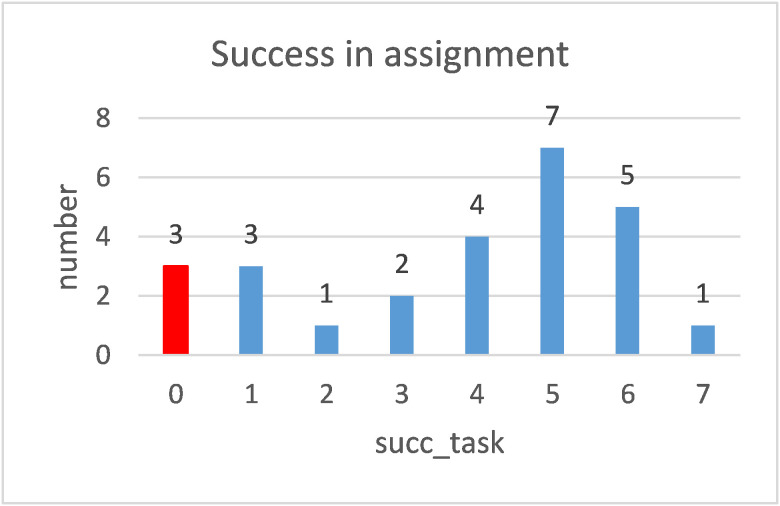
Plot of the distribution of the number of students whose successfully solved tasks

The red column in Figure 2 stands for the students who have not completed even one task correctly.

Dependency between duration time in the seven tasks and total success in the assignment is obtained by non-parametric Spearman coefficient. At first, a new factor including all seven durations is computed using the Factor analysis. Resulting correlation coefficient is 0.54 what could be mentioned as a significant dependency, because also null hypothesis about zero correlation value was rejected (significance = 0.0042). Higher duration time in overall assignment causes higher success.

We compute a percentage success of all students in all assignments (number of successfully solved tasks divided by the total number of tasks). We compare these values for both genders, for the talented students and the standard pupil population.

The Wilcoxon’s nonparametric test was applied to obtain a significance difference. The aim of the Wilcoxon's test is to find out if there is a significant difference between students in the success rate performed by boys and girls. It is usually used when the measured variable (success rate) is not normally divided. The P-level is greater than the significance level of 0.05, we do not reject the null hypothesis, and therefore higher average (percentage) success for male students is not significant (see Table 4).

**Table 4.** Gender distribution of students and results of the Wilcoxon test

group	count	Median	Wilcoxon test	
female	14	57.1	Z-Value	P-Level
male	12	71.4	1.7256	0.084421

**Table 5.** Distribution of standard and gifted students and results of the Wilcoxon test

group	count	Median	Wilcoxon test	
standard=0	22	57.1	Z-Value	P-Level
gifted=1	4	71.4	1.2644	0.206096

The P-level in the same test for the difference of success rate between the talented students and the standard pupil population was also greater than the significance level (0.05), and therefore higher success rate for gifted students is not significant (see Table 5).

There are two ways how to find out if the student’s talent (binary variable) depends on some of the mentioned 65 variables; the discriminant analysis (DA) and the logistic regression (LR). Both methods have the same goal, i.e. to find out a rule how to categorise objects (students) into the groups (talented or not talented) based on other measured variables (binary or numerical). The main difference between these approaches is that DA is more sensitive to the measured data distribution requirement than LR (29).

**Table 6.** Discriminant analysis brief report

variable	standard	gifted
constant	-245.48	-368.08
fc1	0.41	0.51
sl2	-0.22	-0.30
pv3	0.09	0.12
fs4	204.87	249.36
sl4	1.63	1.98
pv4	-0.76	-0.91
cl5	-7.87E-04	-9.87E-04

At the earliest, we applied DA because of the basic regression requirement (the number of the 65 variables has to be smaller than the number of the objects: 26).

**Table 7.** Discriminant analysis prediction success

	predicted		
real	standard	gifted	Total
standard	22	0	22
gifted	0	4	4
Total	22	4	26

The discriminant analysis selected these students’ features as significant to discriminate students on more talented and less talented (see Table 6): gaze fixation count in task No. 1 (fc1), gaze average saccade length in task No. 2 (sl2), gaze path velocity (pv3) of task No. 3, gaze fixation per second (fs4), gaze average saccade length (sl4), gaze path velocity (pv4) of task No. 4, and gaze fixation connection length (cl5) of task No. 5. If we want to know whether another student is rather talented or not, we measure the variables given in Table 6 for the given student, and put the values to a linear equation with parameters for the smaller talent (col “standard”) and the greater talent (col “gifted”), and the greater result value to categorize the student. Along with the coefficients (Table 6), the discriminant analysis provides the table of the success rate of students’ talent prediction (Table 7). We can see that talent of all students was predicted successfully (four more talented and twenty-two less talented).

**Table 8.** Parameters of the logistic regression

variable	Coefficient b(i)
intercept	-133.1798
pv3	0.0383
fs4	30.1339
fc1	0.0980

The logistic regression has a similar purpose as the discriminant analysis. It has only one restriction that the number of the observations has to be greater than the number of the variables. Therefore, we took several variables pre-selected by the discriminant analysis (Table 6) and applied the logistic regression. We obtained the subset of the independent variables (Table 8). The reliability of the model is measured by index R²=0.92 (maximal value is 1). It is clear that three variables out of seven used in DA were cut out.

The quality of the prediction of the student talent remains the same as for discriminant analysis (see Table 7). If we want to apply the model of the logistic regression on a new student (and get a decision about his/her talent), we only put the measured data into a known logistic formula with the parameters from Table 8. The value of probability is provided and then (standardly) the value over 0.5 denotes a more talented student, and vice versa.

**Table 9.** Some of the values of the greatest difference among the students

variable	mean	minimum	maximum	Krr (1), 71
cl6	22583.7	4138.6	74270.2	74270.2
fc5	74.3	15.0	348.0	15.0
fs1	2.9	2.0	3.4	2.0
fs2	2.8	2.1	3.3	2.1
fs5	2.6	1.4	3.9	1.4
fs7	2.6	1.4	3.5	1.4
pv1	851.3	311.6	2766.8	2766.8
pv2	604.1	342.6	1610.1	1610.1
pv3	676.7	194.6	1887.7	1887.7
pv4	622.2	297.0	2399.8	2399.8
pv5	662.3	331.5	1744.2	1744.2
pv6	580.1	183.7	1630.7	1630.7
pv7	649.3	259.5	1383.1	1383.1
sl1	317.7	130.5	1423.6	1423.6
sl2	228.6	112.4	796.6	796.6
sl3	248.7	89.5	918.4	918.4
sl4	255.0	119.7	1249.0	1249.0
sl5	282.2	138.5	1331.7	1331.7
sl6	228.3	88.0	750.2	750.2
sl7	283.2	107.1	1077.6	1077.6
sv1	3.0	1.8	6.7	6.7
sv4	2.9	2.0	4.2	4.2

Furthermore, we wanted to know if there is some possibility to resolve students into some groups (clusters) based on the previously selected variables (fc1 – gaze fixations count No. 1, pv3 – gaze path velocity No. 3, and fs4 – gaze fixations per second No. 4).

**Fig. 3:**
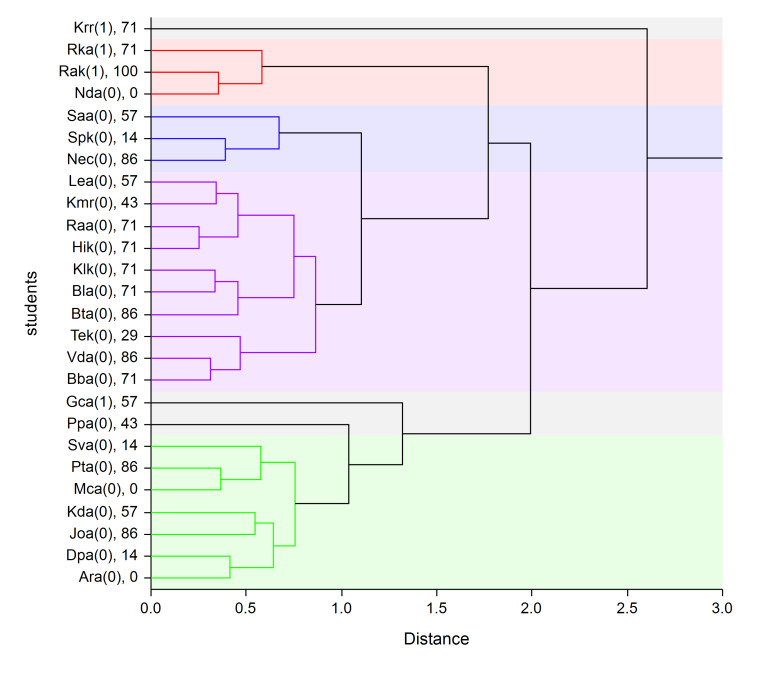
Dendrogram of children with talent and percentage success. Notice: The axis “X“ displays the dissimilarity of an object (distance). A simple average grouping method is applied on variables selected by logistic regression: pv3, fs4, fc1.

There are two traditional approaches how to determine clusters of similar observations - the hierarchical and the non-hierarchical approaches. The first hierarchical cluster analysis was applied to students using only the selected 3 variables (Table 8). We applied a technique called the simple average (weighted per group) and the resulting graph (dendrogram) is in Figure 3.

The sense of a dendrogram is simple. The most similar observation objects (students) are linked together at first (more left in our case), i.e. students abbreviated “Raa” and “Hik” are the most similar in the whole data set (both are less talented / standard students – “0”, and both have the same success – 71 %).

When we focused on gifted students (“1” in brackets), at first the students and “Rak” and “Nda” are linked. Surprisingly, “Rak” is the most successful (and gifted) student in our experiment and “Nda” is standard student with zero success. This pair is later joined with further gifted “Rka”. A big cluster with gifted student (“Gca”) united at the end is joined with mentioned gifted students in final stage, and the last joining (talented) student is “Krr”. This student is the most different student in our experiment.

These students differ in values measured by the Eye-tracker, which is obvious from Table 9, where mean minimal and maximal values of some variables are compared with the values measured for a given student.

No. 6, the longest path velocity for all tasks, the greatest average saccade length for all tasks, the greatest average saccade velocity for tasks No. 1 and No. 4. On the other side, the least scanpath length count is obvious in task No. 5, and the fixations per second in tas ks No. 1, No. 2, No. 5 and No. 7.

The same problem should be solved by the non-hierarchical clustering method, the so-called k-means. The main difference is that the number of the clusters has to be known before the analysis, and in our case there were two of them. We applied the k-means algorithm (to classify the students into two groups), using only three variables detected by the logistic regression (Table 8). Students are divided into two clusters where 3 out of four talented students are in cluster “2” (including student “Krr”) and the last talented “Gca” is in cluster “1”. A difference between average percentage success in two groups (based on k-means method) is relatively big, 63.5 % for students labelled “2” (where all gifted student are included) and 33.9 % for student-group “1”.

**Fig. 4:**
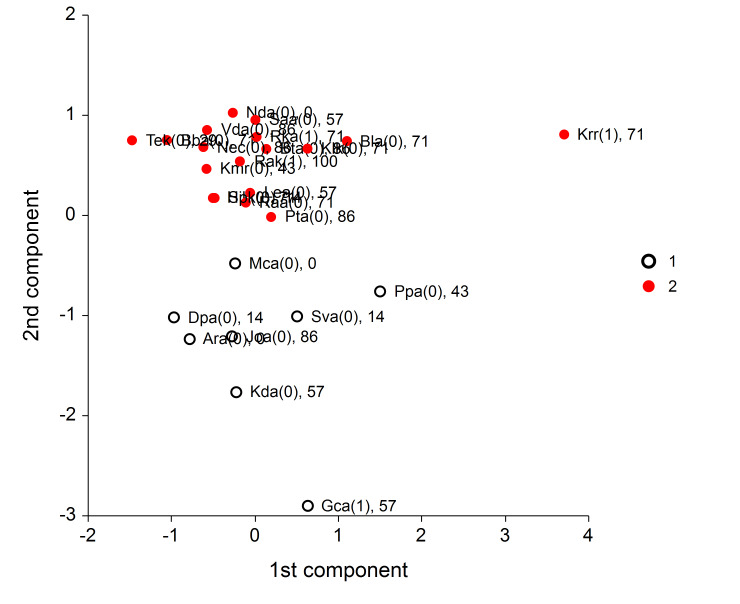
Scatter plot for k-means algorithm describing differences among children

For better understanding of the real differences between students, the four original variables are transformed into two new components (using the Principal component analysis), and these components serve to make the following scatter plot in Figure 4.

We can see that three out of four talented students rather belong to one group (red points of the cluster “2”), while the student “Gca” belongs to the cluster “1”, in which rather worse assignment success was achieved.

Summarizing it, no measured eye-features in the solution of the assignments resulted in any significant student talent differentiation. Nevertheless, the analysis shows some potentially successful ideas in the field of students’ behaviour and their eyes movement.

We are aware that this research is one of the pilot studies in this area, therefore the subsequent validation of results and their generalization will be a task for the next comparative studies and experiments.

## Discussion

The results in Table 10 showed that only one respondent (a male student) had solved all the tasks correctly. Therefore, his AOI solution approach is taken as the standard, with which the similar segments in experimental data are compared within the group of gifted students, as the obtained clusters do suggest a similar problem-solving procedure. For the purpose of an overview, we present Table 10. It contains an analysis of the solution success rate in all seven tasks, based on a simple expression of the number of errors, and on the number of errors in form of the percentage rate – the percentage success rate of the respondents (cf. Table 10).

The correct answers are indicated in bold, the incorrect in italics. We considered any unanswered question (“I do not know”) as a mistake. It is clear from Table 10 that only five respondents made no, or one, mistake. We may consider these students successful, but without comparing the similarity of segments in their data, we cannot consider them gifted. It is necessary to take into account the fact that only about 3% of the current student population in Czechia can be ranked in the gifted students (Ministry of Education, Youth and Sports of the Czech Republic). This means, that in general we can expect at least one gifted student in a sample of 26 respondents. In our research, it was more precisely 0.78 of the “gifted student”.

Analogically, it is also possible to think about the unsuccessful students, but this topic was not the point of our research. We were interested in how the strategies for solving the assigned tasks differ for the students of standard pupil population from the talented students. That is, how the students proceeded in studying the task assignments from the monitor screen, how they continued working with a given assignment, and whether the layout of schemes, tables and application images influenced the correctness of their solutions.

**Table 10.** The overview of the success rate by all respondents in task solving

number of errors	success rate [%]	number of respondents	respondent representation [%]
0	100	1	3.8
1	85.7	4	15.4
2	71.4	7	26.9
3	57.1	5	19.2
4	42.9	2	7.7
5	28.6	1	3.8
6	14.3	3	11.5
7	0	3	11.5
total		26	100

In this respect, we used the demarcation of AOI, but also the time, during which the respondents remained in given AOI, as well as the sequence of their eye-movements (how they orient themselves among various AOI), the time for solving their individual tasks, and the solving time for all the tasks (the total time that respondents needed for a given solution).

The outputs we obtained using the Gazepoint eye-tracking system were complemented by two other research methods - the structured questionnaire survey and the controlled interview with the respondents. As mentioned above, a group of 26 respondents aged 15-16 was addressed.

The research took place in the fourth class of a secondary (Czech grammar) school (“quarta” of a lower “gymnasium”) in June 2017.

The youngest respondent was a 14-year-old boy and the youngest girl was 15. The oldest boy and girl were 16 years old.

**Fig. 5:**
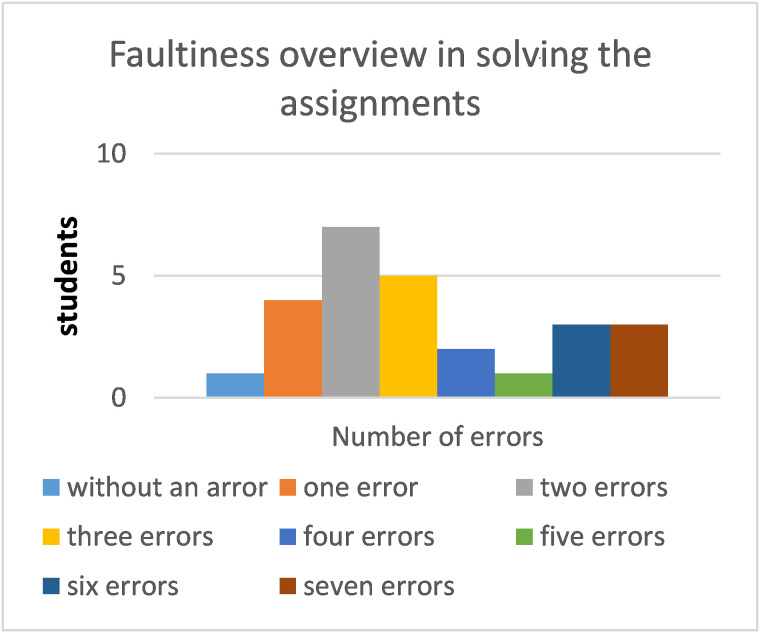
Faultiness overview in solving the assignments

The least number of errors (0 errors) was recorded by a male respondent. The greatest number of errors was recorded by three girls (two of them answered wrongly all questions, and one answered wrongly all questions but she omitted one).

In work with the assignments, lithium “Li” (76.9% of correct answers) became the most successfully identified element although it was assigned as the last task without any hint image. There were two elements with the least success rate, silicon “Si” and gold “Au” (both were identified by respondents at the same percentage rate of 34.6%). From the data obtained, it is also visible that seven female students did not answer a single question, and one girl did not answer more than one question. The boys answered all questions.

By analysis of the answers in the questionnaire (see appendix 3), we have obtained the following findings.

In assignment at first I was attracted by: a - 8 (62%), b - 6 (46%), c - 1 (8%)

a)
  Model of an atom of an element
b)
  Periodic system of elements
c)
  Hint image

**Fig. 6:**
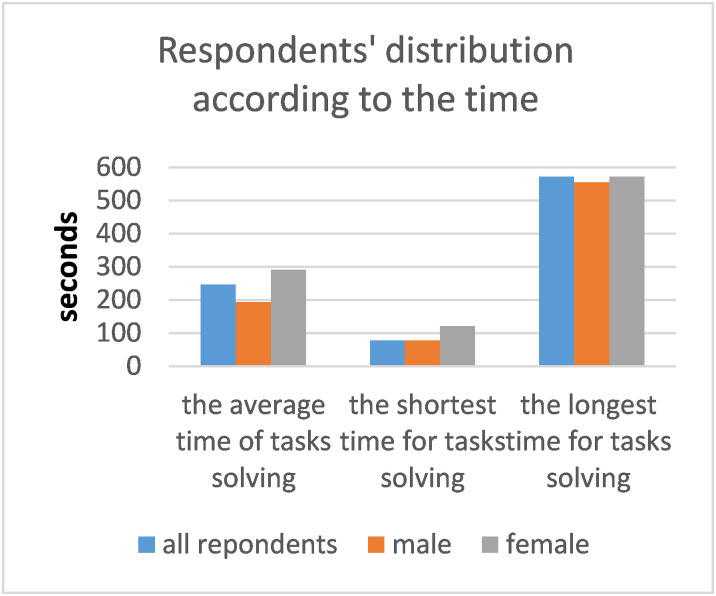
Distribution of respondents according to the time of solving tasks in seconds

To find out which chemical element is concerned in the given task, important for me was: a - 10 (77%), b - 4 (31%), c - 7 (54%), d - 4 (31%)

a)
  Model of an atom of an element
b)
  Hint image
c)
  Periodic system of elements
d)
  Other option (write) .............................................

I did not notice the hint image because: c - 6 (46%), d - 7 (54%)

a)
  It was of a small size
b)
  It was located in an inappropriate place
c)
  I have overlooked it
d)
  Other option (write) ............................................. ..

Answers ad d):

-
  I noticed it
-
  I noticed two or three, not the rest
-
  I did not consider at first, then it turned to be quite important
-
  I did not notice it, so I do not know why I have not noticed it
-
  I thought it was a project mark

If I noticed the hint image, it would help me solving the task: a - 9 (69%), b - 2 (15%)

a)
  Yes
b)
  No.

Answer: “I do not know because I didn’t notice it, and I do not know what was in it” (8%). One questionnaire without answer (8%)

If I could go back to previous assignments, I assume that I would have solved them better:

a)
  Yes, 10 (77%),
b)
  No, 3 (23%)

Underline – while solving the tasks, I realised versus I did not realise that the number of electrons in the electron cloud of the element in the basic state is equal to its proton number.

-
  Realised, 10 (77%)
-
  Not realised, 3 (23%)

## Conclusion

Primarily in our research, we were interested whether the assigned tasks make it to trace the differences in task solutions delivered by the gifted students and the standard pupil population (30).

We analysed how the students followed individual zones that were selected as the areas of interest (AOI). In experimental images we highlighted the AOI and observed if the students look at them. The highlighted AOI were the key factors for successful resolution of the assigned tasks. We found out that information in the AOI was quite used by the gifted students.

It would be possible to study also other aspects, e.g. applying data from OGAMA into ScanGraph (31) that analyses gaze transitions between AOI and compare them with sequence of the mostly gifted respondent. We assume that the features of the ScanGraph will be used in our future studies.

We evaluated how the students proceeded in observing the tasks on the monitor screen throughout the process of solving them, for how long they stayed fixed with eyes on given AOI, in what order they visited given AOI with eye-sight, and how this all affected the success of their solution. In the group of nominated gifted students, we confirmed their mutual similarities, and we correctly detected two gifted students who did not appear in the original nomination. We also followed other contexts that obtained data offered:

-
  success rate of students by gender (higher percentage success for male students was not significant),
-
  distribution of the number of students depending on the successfully solved tasks,
-
  percentage of the success rate of all students in all assignments - we compared these values both in terms of gender and in terms of the talented and the standard students,
-
  relation between the total duration and the total percentage success.

The correlation between the total percentage success and factor made on the duration time of the solution from all of the seven tasks was calculated. This correlation could show us, among other things, whether the duration time of solution to a particular task has any influence on the total success rate of the tasks solving (or not). Provided coefficient (0.54) enables us to find out that the longer duration time of overall assignment solving, the higher total percentage success. We also proved that the most successful respondent within the group of gifted students needed a very short time to successfully solve the assigned tasks.

We constituted a question whether talent depends on some further features that can be measured by the Gazepoint eye-tracking device. In context of the technology used and the assignments, we had eight variables (attributes), each of which we measured seven-times independently of each task (i.e. 56 independent variables). In addition, we could work with the binary variable for the success rate in each “task-gender” of students and the total percentage success. We used thus two ways to find out whether students’ talent depends on some of these variables, the discriminant analysis and the logistic regression. The discriminant analysis has selected the following factors significant in discrimination of students on the less and more talented. These factors include: fixation time (fc1), observation of the average duration time of volume in task No. 2 (sl2), fixation per second (fs4), average duration time of the volume (sl4), velocity of track observation in task No. 4 (pv4).

The discriminant analysis has shown that all gifted students were in advance correctly identified. Therefore, the Gazepoint eye-tracking system can be successfully used to verify the teacher’s prediction in relation to the talent of students. Nevertheless, the analysis has shown some potentially successful ideas in the areas of students’ behaviour and their eye-movements. The logistic regression has a similar purpose as the discriminant analysis. There is only one limitation that the number of observations has to be greater than the number of variables. Therefore, we used seven variables pre-selected by the discriminant analysis, and afterwards we applied the logistic regression. We have found out that the quality of the talent prediction remains the same and the number of variables necessary to distinguish talented and standard students decrease to three.

To supplement the research, we were interested in whether the students worked with the hint images (whether they noticed them) in the course of solving the assigned tasks. We found answer to this question from the results of the questionnaire survey. The hint image was included in almost all assignments for several reasons:

-
  to help students better orient themselves in the assignment,
-
  to enable students to make use of knowledge related to concrete elements,
-
  and to help students resolve a given assignment faster.

We assumed that:

-
  gifted students will notice the hint image earlier than standard students,
-
  gifted students will actively use the hint image for task solving,
-
  duration time of tasks solving will be shorter for gifted students using an interactive image than for standard students.

We even intentionally placed hint images onto different areas of the monitor screen. First, the hint images were twice placed onto the same area of the screen (the right bottom corner). Then the positions of the hint images were changed over the entire area of the monitor screen. For the last element, lithium, we did not intentionally include the hint image onto the screen. We wanted to find out if the students start looking actively for the hint image, which did not happen. Disappointing as well as surprising findings for us were that our assumptions for working with the hint image were not correct. 46% of the respondents simply ignored the hint image.

On the other hand, 69% of the students revealed that if they had noticed the hint image, it would have helped them solve the task. And if they could have gone back to the previous assignments, 77% of the respondents would have expected better resolution of the tasks. We find our findings very interesting. They mean that students do not look at the full screen (perceiving the assignment on the entire screen), but only at its noticeable parts. Therefore, we had a question whether the students can work with the full screen area (with all information)? Regardless of their talents, they certainly can not.

What does it mean from the point of view of the field-didactic interpretations? It confirms that students in Czech schools are mainly focused on performance. Immediately, when the students saw the assignment, they began to actively solve the tasks and did not basically occupy themselves with the surroundings of the main parts of the assignments (element diagram, periodic system of elements). Therefore, the question for the pedagogical public is whether the student’s focus on fast performance is correct. And if it is not (or not always), how we can set it right so that the outcome is beneficial for the whole educational process?  

## Ethics and Conflict of Interest

The author(s) declare(s) that the contents of the article are in agreement with the ethics described in http://biblio.unibe.ch/portale/elibrary/BOP/jemr/ethics.html and that there is no conflict of interest regarding the publication of this paper.

## Acknowledgements

This paper is the result of a pilot eye-tracking study at the newly established Centre for Research on Natural Science Education and Talent-Management, Faculty of Science of the University of Ostrava, Czechia.

We express gratitude to Mgr. Vladimír Bradáč, Ph.D. and Mgr. Stanislav Popelka, Ph.D. for useful comments.
